# Supplementary material for: Frequency, kinetics and determinants of viable SARS-CoV-2 in bioaerosols from ambulatory COVID-19 patients infected with the Beta, Delta or Omicron variants
Source: Nat Commun. 2024 Mar 5;15:2003. doi: 10.1038/s41467-024-45400-1 (PMC10914788; doi:10.1038/s41467-024-45400-1)
Supplement: Supplementary file 1 — Supplementary Information [file 41467_2024_45400_MOESM1_ESM.pdf]

ONLINE SUPPLEMENT

SUPPLEMENTARY TABLES

**Gene** / *Mutation* ‡	**Culture negative aerosol (n=12)**	**Culture positive aerosol (n=32)**	**Gene** / *Mutation* ‡	**Culture negative aerosol (n=12)**	**Culture positive aerosol (n=32)**	**Gene** / *Mutation* ‡	**Culture negative aerosol (n=12)**	**Culture positive aerosol (n=32)**
**nsp4**			**nsp16**			**N**		
A298ADGV	1		*V289F*		1	*N213I*		1
L90LF		1	**Plpro**			*D63DAGV*	1	
**nsp6**			*Q383H*		1	*G215GCRS*	1	
F228FILV	1		*V1770I*		1	*Y298Y**	1	
**nsp13**			*G1447X*		1	**M**		
P593DE	1		*Y1448X*		1	*H210X*	1	
R594X	1		**RdRp**			*S211IRT*	1	
P593PLQR		1	*D445DHNY*	1		*S213GR*	1	
Q601QLPR		1	*V473VFIL*	1		*G192GADV*	1	
A267T		1	*G503GADV*	1		*A194APST*	1	
**nsp14**			**Spike**			*I161IM*	1	
*K457K*EQ*	1		*V308I*		1	*K162K*EQ*	1	
S112SCFY	1		*G744S*		1	*Y199YCFS*	1	
*W385W*LS*	1		*M153V*		1	*S184DE*		1
A394V		1	*E180E8KQ*	1		*Q185X*		1
*G6G*R*		1	*D1084DHNY*	1		*R186X*		1
L54LFIV		1	*G75S*	1		*D3N*		1
*E453E*KQ*		1	*N658S*	1	2			
S454SAPT		1	**ORF3a**					
G456GAEV		1	*I20IFLV*		1			
S470SCFY		1	*H78HDNY*		1			
A471ADGV		1	**ORF8**					
T472TKMR		1	*V81VFIL*	1				
C473CFSY		1						
T475TIKR		1						

Table S1: Unique mutations (found in <0.01% of genomes) and their association with aerosol culture status

‡ unusual mutation as having a global prevalence below 0.01%.

Table S2: Accuracy of nasopharyngeal swab Ct as a proxy of aerosol culture positivity

Sensitivity (95% CI)	Specificity (95% CI)	Positive Predictive Value (95% CI)	Negative Predictive Value (95% CI)	Accuracy (95% CI)	Ct cut-off
96.77 (83-100)	46.15 (19-75)	81.08 (65-92)	85.71 (42-100)	81.82 (67-92)	26.7
90.32 (74-98)	61.54 (32-86)	84.85 (68-95)	72.73 (39-94)	81.82 (67-92)	22.6
80.65 (63-93)	61.54 (32-86)	83.33 (65-94)	57.14 (29-82)	75.00 (60-87)	21.8
70.97 (52-86)	61.54 (32-86)	81.48 (62-94)	47.06 (23-72)	68.18 (52-81)	19.5
61.29 (42-78)	84.62 (55-98)	90.48 (70-99)	47.83 (27-69)	68.18 (52-81)	17.8
48.39 (30-67)	84.62 (55-98)	88.24 (64-99)	40.74 (22-61)	59.09 (43-74)	17.0
38.71 (22-58)	92.31 (64-100)	92.31 (64-100)	38.71 (22-58)	54.55 (39-70)	16.3
29.03 (14-48)	92.31 (64-100)	90.00 (56-100)	35.29 (20-54)	47.73 (32-63)	14.6
19.35 (7-37)	92.31 (64-100)	85.71 (42-100)	32.43 (18-50)	40.91 (26-57)	13.6
9.68 (2-26)	100 (75-100)	100.00 (29-100)	31.71 (18-48)	36.36 (22-52)	11.4

Table S3: Association between aerosol culture positivity and nasopharyngeal swab, cough tubing or saliva culture status. The Fisher's exact test was used for comparisons between groups.

	Culture negative aerosol	Culture positive aerosol (<10 µm)	p-value	Culture positive aerosol (<5 µm)	p-value
Culture positive NPS	19 (45)	40 (87)	p<0.0001	31 (94)	p<0.0001
Culture negative NPS	23 (55)	6 (13)		2 (6)	
Culture positive Cough Tubing	7 (17)	40 (87)	p<0.0001	30 (91)	p<0.0001
Culture negative Cough Tubing	35 (83)	6 (13)		3 (9)	
Culture positive Saliva	8 (21)	24 (55)	p=0.003	19 (61)	p=0.001
Culture negative Saliva	30 (79)	20 (45)		12 (39)	

Table S4: Accuracy of nasopharyngeal swab Ct as a proxy for identification of highly infectious cases

Sensitivity (95% CI)	Specificity (95% CI)	Positive Predictive Value (95% CI)	Negative Predictive Value (95% CI)	Accuracy (95% CI)	Ct cut-off
94.74 (74-100)	58.33 (28-85)	78.26 (56-93)	87.50 (47-100)	80.65 (63-93)	22.6
89.47 (28-85)	58.33 (28-85)	77.27 (55-92)	77.78 (40-97)	77.42 (59-90)	22.4
78.95 (54-94)	58.33 (28-85)	75 (51-91)	63.64 (31-89)	70.97 (52-86)	21.9
68.42 (43-87)	58.33 (28-85)	72.22 (47-90)	53.85 (25-81)	64.52 (45-81)	19.5
57.89 (34-80)	83.33 (52-98)	84.62 (55-98)	55.56 (31-78)	67.74 (49-83)	17.1
47.37 (24-71)	83.33 (52-98)	81.82 (48-98)	50.00 (27-73)	61.29 (42-78)	16.9
42.11 (20-67)	91.67 (62-100)	88.89 (52-100)	50.00 (28-72)	61.29 (42-78)	16.6
31.58 (13-57)	91.67 (62-100)	85.71 (42-100)	45.83 (26-67)	54.84 (36-73)	14.6
21.05 (6-46)	91.67 (62-100)	80.00 (28-100)	42.31 (23-63)	48.39 (30-67)	13.8
10.53 (1-33)	91.67 (62-100)	66.67 (9-99)	39.29 (22-59)	41.94 (25-61)	13.6

Table S5: DE analysis: CASS positive / CASS negative

	CASS positive / CASS negative (FDR<0.05)	CASS positive / CASS negative (p<0.05)
Up	11	1735
Down	9	1281
Not significant	17334	14338
Total	17354	17354

SUPPLEMENTARY FIGURES

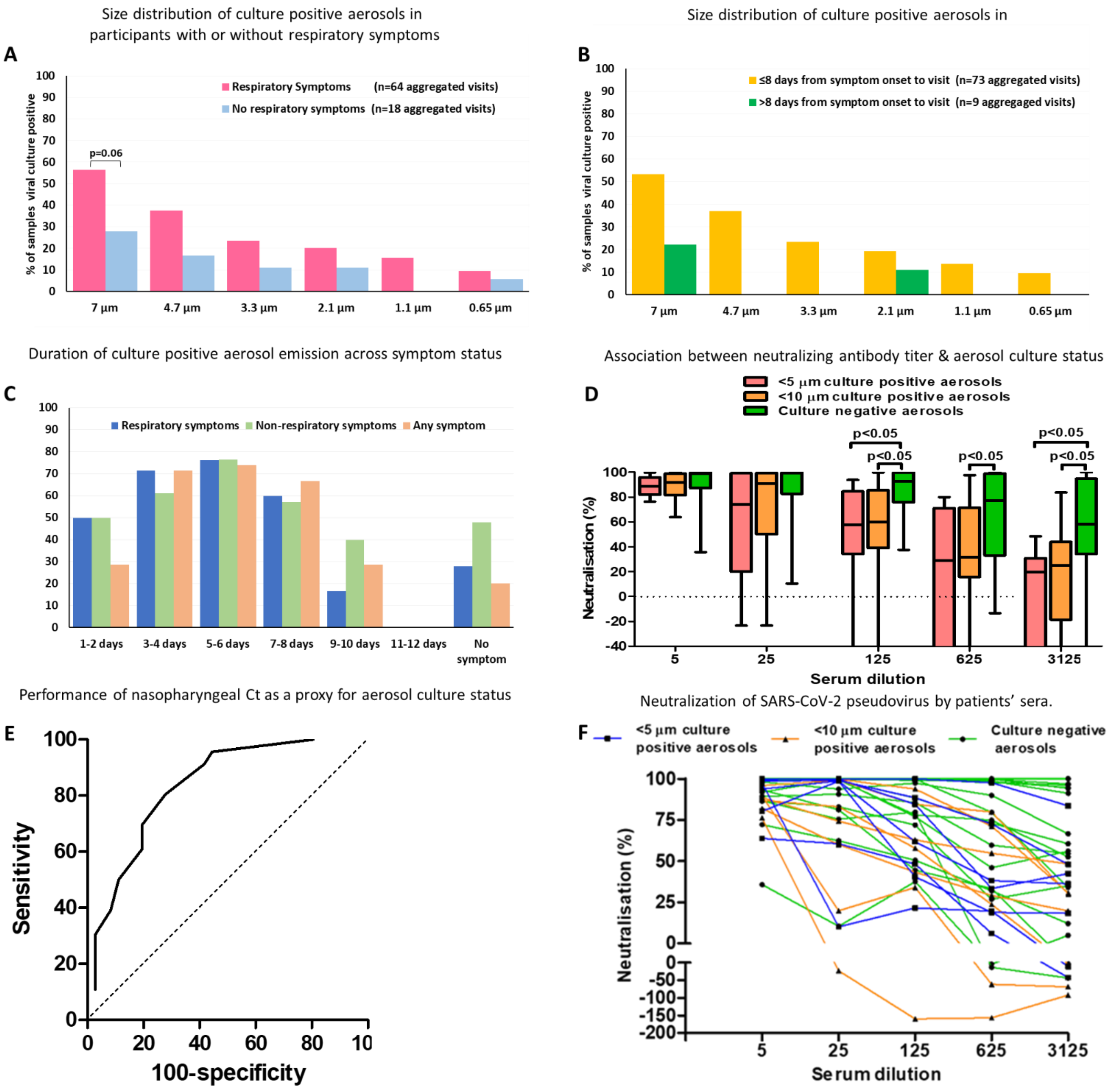

Supplementary Figure 1: (A) Proportion of size-fractionated aerosol samples that were from participants with (■) or without (■) respiratory symptoms that were culture-positive (visits aggregated, i.e visit 1 + visit 2 = 82 visits in total). **(B)** Proportion of size-fractionated aerosol samples that were from participants with duration from symptom onset to sampling ≤8 days (■) or >8 days (■) (visits aggregated, i.e visit 1 + visit 2 = 82 visits in total). **(C)** Temporal pattern of culture positive aerosol

(<10 µm) emission in cases with respiratory symptoms (■), non-respiratory symptoms (■) or any symptom (■). **(D) Neutralization potential:** across aerosol culture status, expanded to include further aerosol size grading and more serum dilution series. **(E) ROC for nasopharyngeal Ct value:** as a proxy for aerosol culture positivity (generated from values in table S2). **(F) Neutralization activity**: in aerosol culture positive and negative persons (individual plots).

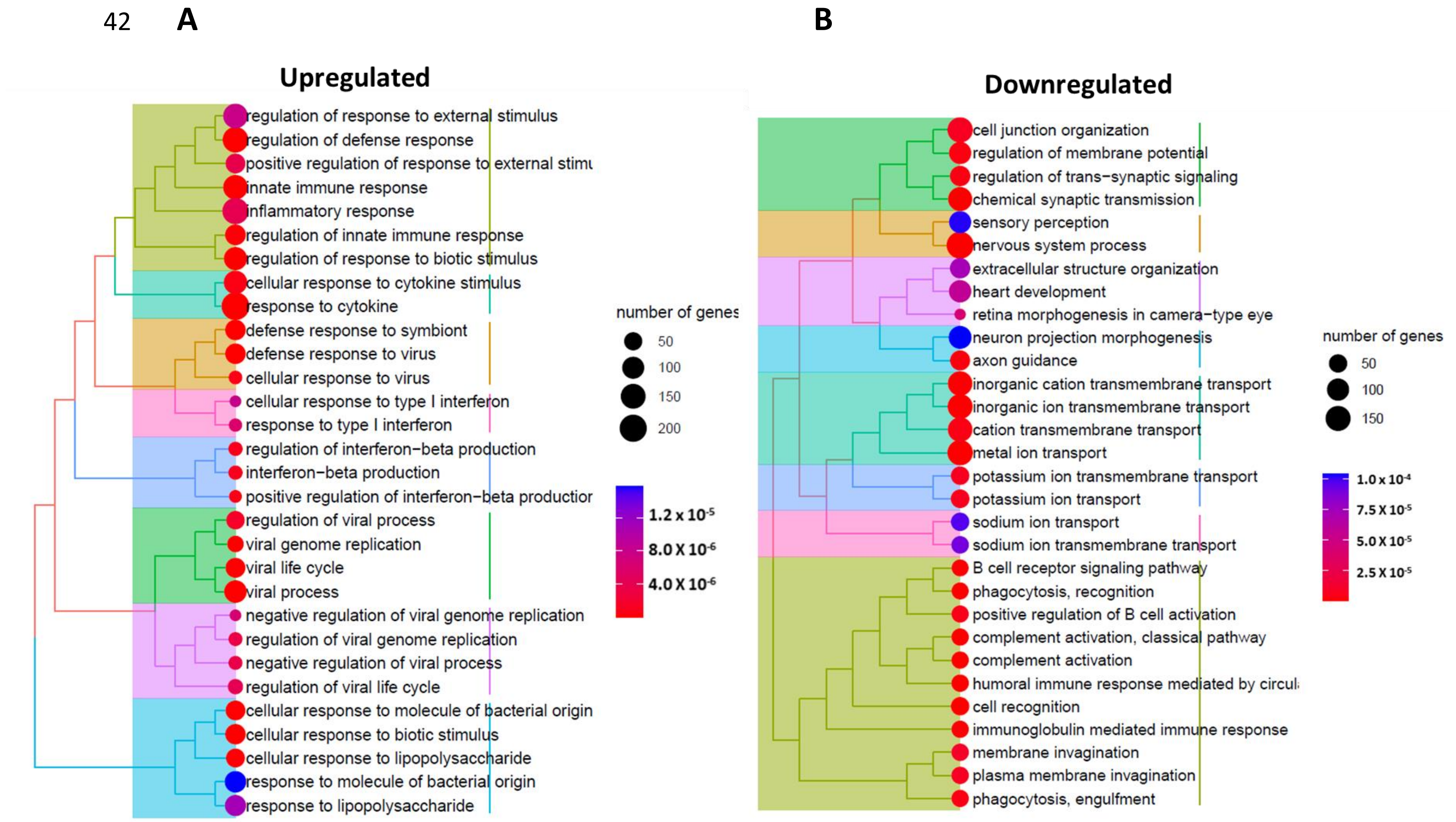

Supplementary Figure 2: Tree plots of gene set enrichment analysis results: illustrating the 30 most **(A)** enriched and **(B)** suppressed gene ontology biological process pathways when comparing CASS-positive to CASS-negative individuals. Gene sets with high similarity are clustered (hierarchical) into subtrees to reduce the complexity of the results allowing improved interpretability. For the activated pathways clusters include innate/inflammatory responses and response to cytokines.

A

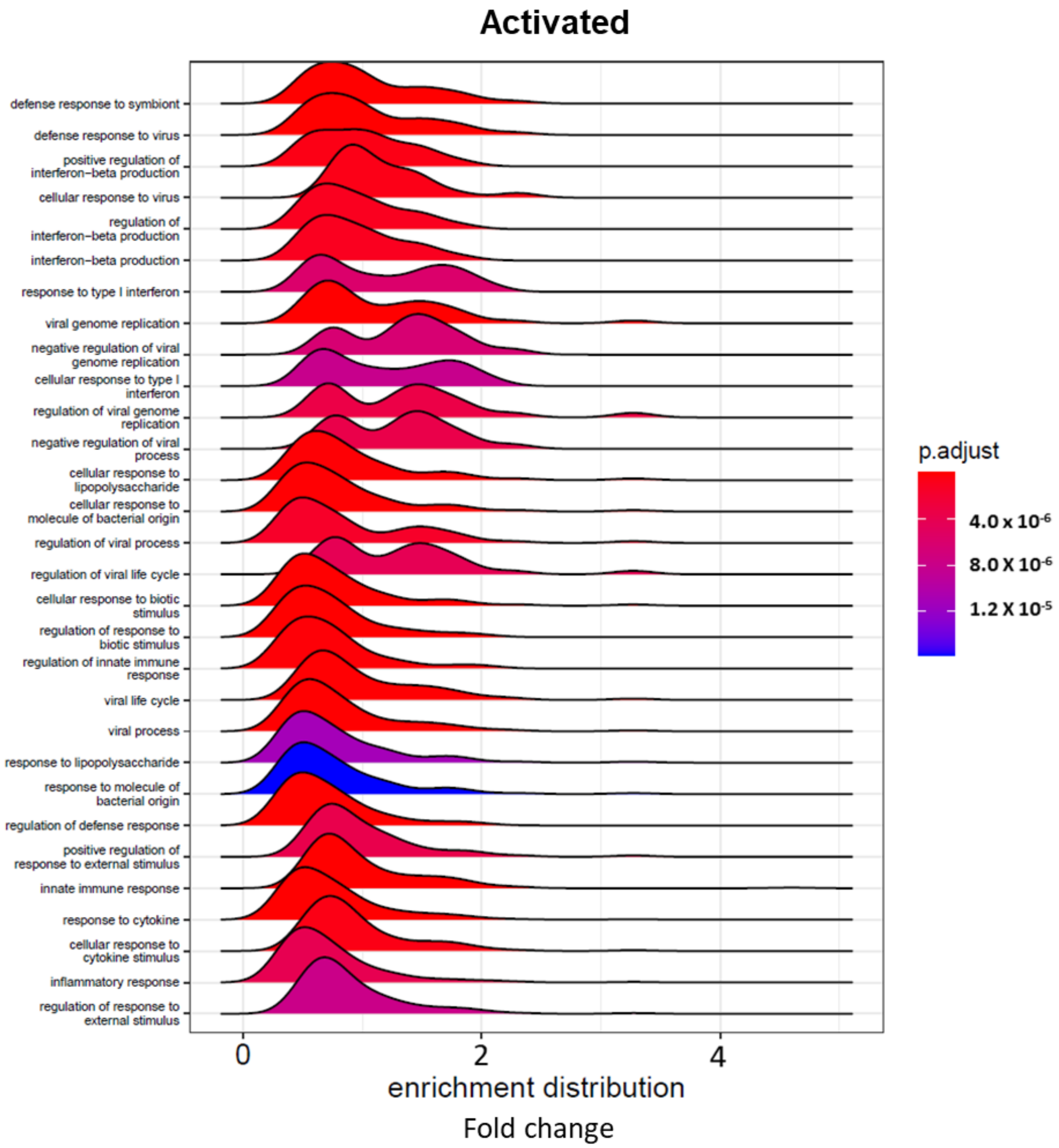
Activated
defense response to symbiont
defense response to virus
positive regulation of interferon−beta production
cellular response to virus
regulation of interferon−beta production
interferon−beta production
response to type I interferon
viral genome replication
negative regulation of viral genome replication
cellular response to type I interferon
regulation of viral genome replication
negative regulation of viral process
cellular response to lipopolysaccharide
cellular response to molecule of bacterial origin
regulation of viral process
regulation of viral life cycle
cellular response to biotic stimulus
regulation of response to biotic stimulus
regulation of innate immune response
viral life cycle
viral process
response to lipopolysaccharide
response to molecule of bacterial origin
regulation of defense response
positive regulation of response to external stimulus
innate immune response
response to cytokine
cellular response to cytokine stimulus
inflammatory response
regulation of response to external stimulus
0
2
4
enrichment distribution
Fold change
p.adjust
4.0 x 10-6
8.0 X 10-6
1.2 X 10-5

B

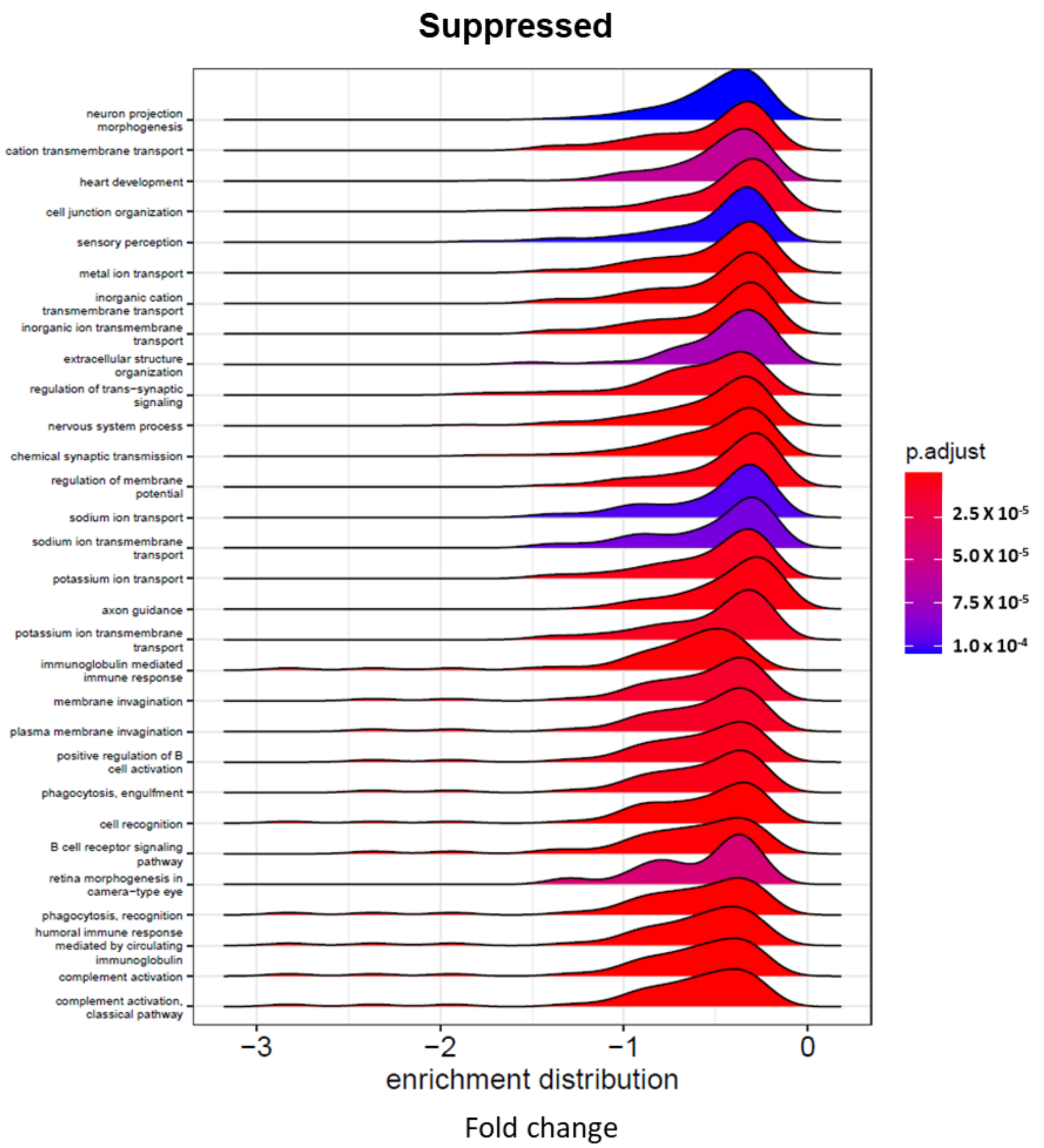

Supplementary Figure 3: Ridgeplots of gene set enrichment analysis results: illustrating the 30 most **(A)** enriched and **(B)** suppressed gene ontology biological process pathways when comparing CASS-positive to CASS-negative individuals. Pathway are listed on the y-axis while log2 fold-change is on the x-axis. The density plot for each pathway represents the fold-change distribution for the core enriched genes in each pathway. In summary, activated pathways in CASS- positive individuals include responses to biotic stimulus including virus and bacteria, innate and inflammatory immune responses as well as production and responses to cytokines and type I interferon. Pathways that are suppressed in CASS positive individuals include complement and B cell activation, phagocytosis and

immunoglobulin mediated immune responses as well as pathways related to ion transport, development and neuronal sensory perception.

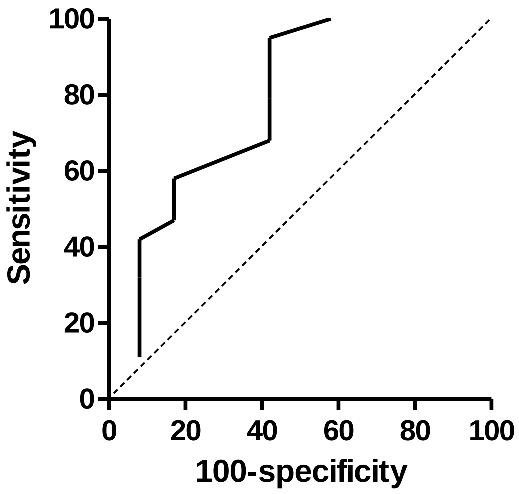

Supplementary Figure 4: ROC for nasopharyngeal Ct as a proxy for identification of probably highly infectious cases (generated from values in table S4).

Table S6: Accession codes for all sequencing data

Participant ID	Genbank Accession ID	Participant ID	Genbank Accession ID
CoV-CASS 001	ON078382	CoV-CASS 023	ON055809
CoV-CASS 002	ON078383	CoV-CASS 024	ON055810
CoV-CASS 003	ON078384	CoV-CASS 025	ON055811
CoV-CASS 004	ON078385	CoV-CASS 026	ON055812
CoV-CASS 005	#	CoV-CASS 027	*
CoV-CASS 006	#	CoV-CASS 028	ON116595
CoV-CASS 007	#	CoV-CASS 029	ON116596
CoV-CASS 008	ON078387	CoV-CASS 030	ON116597
CoV-CASS 009	ON078389	CoV-CASS 031	ON116598
CoV-CASS 010	ON078390	CoV-CASS 032	ON116599
CoV-CASS 011	ON055807	CoV-CASS 033	ON116600
CoV-CASS 012	ON078444	CoV-CASS 034	ON116601
CoV-CASS 013	ON322592	CoV-CASS 035	ON116602
CoV-CASS 014	ON078445	CoV-CASS 036	ON116603
CoV-CASS 015	ON078446	CoV-CASS 037	ON116604
CoV-CASS 016	ON055808	CoV-CASS 039	OQ341824
CoV-CASS 017	ON078447	CoV-CASS 040	^
CoV-CASS 018	ON078448	CoV-CASS 041	OQ341825
CoV-CASS 019	ON078449	CoV-CASS 042	OQ341826
CoV-CASS 020	ON078450	CoV-CASS 043	OQ341827
CoV-CASS 021	ON078451	CoV-CASS 044	OQ341828
CoV-CASS 022	*	CoV-CASS 045	OQ341829

# there is no data, but it is reported as a failed sequence assigned as Beta.

* there is no data, but it is reported as a failed sequence assigned as Delta.

^ wasn’t sequenced because it failed QC upstream of sequencing, but is reported as a failed sequence assigned as Omicron.
