# Supplementary material for: Frequency, kinetics and determinants of viable SARS-CoV-2 in bioaerosols from ambulatory COVID-19 patients infected with the Beta, Delta or Omicron variants
Source: Nat Commun. 2024 Mar 5;15:2003. doi: 10.1038/s41467-024-45400-1 (PMC10914788; doi:10.1038/s41467-024-45400-1)
Supplement: Supplementary file 3 — Reporting Summary [file 41467_2024_45400_MOESM3_ESM.pdf]

Corresponding author(s): Keertan Dheda

Last updated by author(s): Dec 13, 2023

Reporting Summary

Nature Portfolio wishes to improve the reproducibility of the work that we publish. This form provides structure for consistency and transparency in reporting. For further information on Nature Portfolio policies, see our Editorial Policies and the Editorial Policy Checklist.

Statistics

For all statistical analyses, confirm that the following items are present in the figure legend, table legend, main text, or Methods section.

n/a	Confirmed	
☐	☒	The exact sample size (*n*) for each experimental group/condition, given as a discrete number and unit of measurement
☐	☒	A statement on whether measurements were taken from distinct samples or whether the same sample was measured repeatedly
☐	☒	The statistical test(s) used AND whether they are one- or two-sided<br>*Only common tests should be described solely by name; describe more complex techniques in the Methods section.*
☐	☒	A description of all covariates tested
☐	☒	A description of any assumptions or corrections, such as tests of normality and adjustment for multiple comparisons
☐	☒	A full description of the statistical parameters including central tendency (e.g. means) or other basic estimates (e.g. regression coefficient) AND variation (e.g. standard deviation) or associated estimates of uncertainty (e.g. confidence intervals)
☐	☒	For null hypothesis testing, the test statistic (e.g. *F*, *t*, *r*) with confidence intervals, effect sizes, degrees of freedom and *P* value noted<br>*Give P values as exact values whenever suitable.*
☒	☐	For Bayesian analysis, information on the choice of priors and Markov chain Monte Carlo settings
☒	☐	For hierarchical and complex designs, identification of the appropriate level for tests and full reporting of outcomes
☒	☐	Estimates of effect sizes (e.g. Cohen's *d*, Pearson's *r*), indicating how they were calculated

Our web collection on statistics for biologists contains articles on many of the points above.

Software and code

Policy information about availability of computer code

Data collection	Data collection done through the use of paper-based case report forms and data transcribed onto REDCap database.
Data analysis	Statistical analysis was performed using Stata version 17 and GraphPad, Version 9.4.1. Transcriptomic analysis was done using edgeR Version 3.38.4, FastQC program Version 0.11.9, Trim Galore program Version 0.6.10 and R (Version 4.2.3).

For manuscripts utilizing custom algorithms or software that are central to the research but not yet described in published literature, software must be made available to editors and reviewers. We strongly encourage code deposition in a community repository (e.g. GitHub). See the Nature Portfolio guidelines for submitting code & software for further information.

Data

Policy information about availability of data

All manuscripts must include a data availability statement. This statement should provide the following information, where applicable:

- Accession codes, unique identifiers, or web links for publicly available datasets
- A description of any restrictions on data availability
- For clinical datasets or third party data, please ensure that the statement adheres to our policy

Individual participant data will be made available to researchers who provide a protocol that is approved by their respective human research ethics committee. All protocols will be reviewed and approved by the CASS COVID consortium trial steering committee up to five years following publication. A data sharing agreement

(DTA) will need to be concluded between the representatives of the requesting institution and the University of Cape Town Lung Institute. Data sharing requests should be directed to keertan.dheda@uct.ac.za. Accession codes for sequencing done for the study is provided at the end of the submitted manuscript.

Research involving human participants, their data, or biological material

Policy information about studies with human participants or human data. See also policy information about sex, gender (identity/presentation), and sexual orientation and race, ethnicity and racism.

Reporting on sex and gender	The proportion of male and female participants in the study was reported. Sex was a self-reported status by study participants. However, sex was not considered in the study. For the purposes of this study, only male and female sex were categorized. None of the participants refused to provide response for this question.
Reporting on race, ethnicity, or other socially relevant groupings	Race, ethnicity or any grouping not based on biological or clinical status were not considered for analysis.
Population characteristics	Confirmed COVID-19 infected ambulatory participants, enrolled within a maximum of 7 days from symptom onset, or diagnosed as asymptomatic cases through contact tracing. All participants were adults, self-identified sex was reported and a medical history reflecting existing comorbidities and COVID vaccine status was documented.
Recruitment	Participants were screened and recruited through a study evaluating rapid diagnostics for COVID-19 (antigen and antibody point-of-care lateral flow assay. Eligible participants had to be PCR or rapid antigen positive, be within 7 days of symptom onset, ambulatory, have a blood oxygen saturation (O2 SAT) greater than 95 and consent to undergo CASS and provide nasopharyngeal and saliva specimens. A follow-up visit was scheduled for 2-3 days after enrollment and defaulting on follow-up visit did not disqualify the participant from being included into the study. Recruitment into this study did not reflect a selection bias.
Ethics oversight	Ethical approval was obtained from the Human Research Ethics Committee (HREC) of the University of Cape Town (HREC approval number 387/2020). Biosafety approvals were obtained from the Faculty Biosafety Committee of the University of Cape Town (IBC043-2020). Informed consent was obtained from all participants included in this study.

Note that full information on the approval of the study protocol must also be provided in the manuscript.

Field-specific reporting

Please select the one below that is the best fit for your research. If you are not sure, read the appropriate sections before making your selection.

☒ Life sciences ☐ Behavioural & social sciences ☐ Ecological, evolutionary & environmental sciences

For a reference copy of the document with all sections, see nature.com/documents/nr-reporting-summary-flat.pdf

Life sciences study design

All studies must disclose on these points even when the disclosure is negative.

Sample size	As there were no prior sample sets of more than 5 culture aerosol positive participants in any one study, and lack of prior data, we used a prevalence estimate method to calculate sample size. We ascertained that 43 participants would need to be recruited to allow us to estimate a 50% frequency of cough aerosol positivity within a 15% margin of error.
Data exclusions	No participants were excluded from the study. All enrolled participants completed the aerosol sampling procedure without any protocol deviation at their baseline visit and provided the requested nasopharyngeal swab samples.
Replication	Our primary outcome measure was aerosol viral culture positivity. All procedures for viral culture followed the laboratory biosafety guidelines developed and approved by the University of Cape Town (UCT) Institutional Biosafety Committee (IBC) and were conducted in a BSL 3 facility. The viral culture experiments were verified for reproducibility by performing the assay for a total of 30 samples on 2 separate days. Viral culture of nasopharyngeal swab samples (n=6), cough tubing samples (n=5), saliva (n=7) and aerosol samples (n=12), had a combined pooled R squared value of 0.88 ($p<0.0001$), which was indicative of high reproducibility.<br>We have optimised and validated the RTqPCR assay for determination of viral load. RNA was extracted using the TANbead automated nucleic acid extraction system. Extracted RNA was amplified using the Centers for Disease Control and Prevention (CDC) approved nCOV 2019 kit that detects two targets within the nucleocapsid coding region (N1 and N2). An internal control, human RP gene, was also included. A standard curve was generated using serial dilutions of RNA extracted from SARS-CoV-2 viral culture at a known concentration. Both N1 and N2 target copies are quantified by comparing Ct values to the standard curve. These measures ensured standardisation of measurements.
Randomization	Not applicable as this was not a trial and no interventions were being assessed and compared.
Blinding	There was no blinding in the study design. No bias was introduced in data generation or analysis.

Reporting for specific materials, systems and methods

We require information from authors about some types of materials, experimental systems and methods used in many studies. Here, indicate whether each material, system or method listed is relevant to your study. If you are not sure if a list item applies to your research, read the appropriate section before selecting a response.

Materials & experimental systems

n/a	Involved in the study
☒	☐ Antibodies
☐	☒ Eukaryotic cell lines
☒	☐ Palaeontology and archaeology
☒	☐ Animals and other organisms
☒	☐ Clinical data
☒	☐ Dual use research of concern
☒	☐ Plants

Methods

n/a	Involved in the study
☒	☐ ChIP-seq
☒	☐ Flow cytometry
☒	☐ MRI-based neuroimaging

Eukaryotic cell lines

Policy information about cell lines and Sex and Gender in Research

Cell line source(s)	The human lung carcinoma cell line, H1299 H2AZ-clone, was a gift from Dr Alex Sigal, Africa Health Research Institute, Durban, South Africa. The cell line was constructed to over express ACE2. It was originally isolated from a 43 year old white male.
Authentication	The authenticity of the cell line was confirmed by Dr Alex Sigal as described in the following publication: Cele et al., 2021. Nature 593, 142-146.
Mycoplasma contamination	e cell line was routinely tested for mycoplasma by Dr Alex Sigal at the Africa Health Research Institute, Durban, South Africa, using the LookOut Mycoplasma PCR detection Kit (Sigma #MP0035) .
Commonly misidentified lines (See ICLAC register)	Not applicable.

Plants

Seed stocks	*Report on the source of all seed stocks or other plant material used. If applicable, state the seed stock centre and catalogue number. If plant specimens were collected from the field, describe the collection location, date and sampling procedures.*
Novel plant genotypes	*Describe the methods by which all novel plant genotypes were produced. This includes those generated by transgenic approaches, gene editing, chemical/radiation-based mutagenesis and hybridization. For transgenic lines, describe the transformation method, the number of independent lines analyzed and the generation upon which experiments were performed. For gene-edited lines, describe the editor used, the endogenous sequence targeted for editing, the targeting guide RNA sequence (if applicable) and how the editor was applied.*
Authentication	*Describe any authentication procedures for each seed stock used or novel genotype generated. Describe any experiments used to assess the effect of a mutation and, where applicable, how potential secondary effects (e.g. second site T-DNA insertions, mosiacism, off-target gene editing) were examined.*
